# Supplementary material for: Selectivity matters: selective ROCK2 inhibitor ameliorates established liver fibrosis via targeting inflammation, fibrosis, and metabolism
Source: Commun Biol. 2023 Nov 18;6:1176. doi: 10.1038/s42003-023-05552-0 (PMC10657369; doi:10.1038/s42003-023-05552-0)
Supplement: Supplementary file 2 — Supplementary figures [file 42003_2023_5552_MOESM2_ESM.pdf]

Selectivity matters: Highly selective ROCK2 inhibitor ameliorates established liver fibrosis via targeting inflammation, fibrosis, and metabolism.

Alexandra Zanin-Zhorov[1,*], Wei Chen[1,#], Julien Moretti[1,#], Melanie S. Nyuydzefe[1,#], Iris Zhorov[1], Rashmi Munshi[2], Malavika Ghosh[2], Cindy Serdjebi[3], Kelli MacDonald[4], Bruce R. Blazar[5], Melissa Palmer[6] and Samuel D. Waksal[1]

[1]Graviton Bioscience B.V., Amsterdam, Netherlands 1017 CG

[2]Aragen Bioscience Inc., Morgan Hill, CA 95037, USA

[3]Biocellvia 13001 Marseille, France

[4]QIMR Berghofer Medical Research Institute, Brisbane, Australia, 4006

[5]Division of Blood & Marrow Transplant & Cellular Therapies, University of MN, Masonic Cancer Center and Department of Pediatrics, Minneapolis, MN 55455, USA

[6]Liver Consulting LLC, Dix Hills, NY 11746, USA

[*]Corresponding author: Alexandra.zanin-zhorov@gravitoncorp.com

[#]These authors contributed equally to the work.

SUPPLEMENTARY INFORMATION: SUPPLEMENTARY FIGURES 1-5

Supplementary Figure 1. Selective ROCK2 inhibitor GV101 decreased established TAA-induced liver fibrosis.

a

	ROCK2 (IC50 nM)	ROCK1 (IC50 nM)
**GV101**	7.5±7.9	>30000
**KD025**	287±128	>30000
**H1152**	9.5	5
**GSK429286**	9.08	8.17

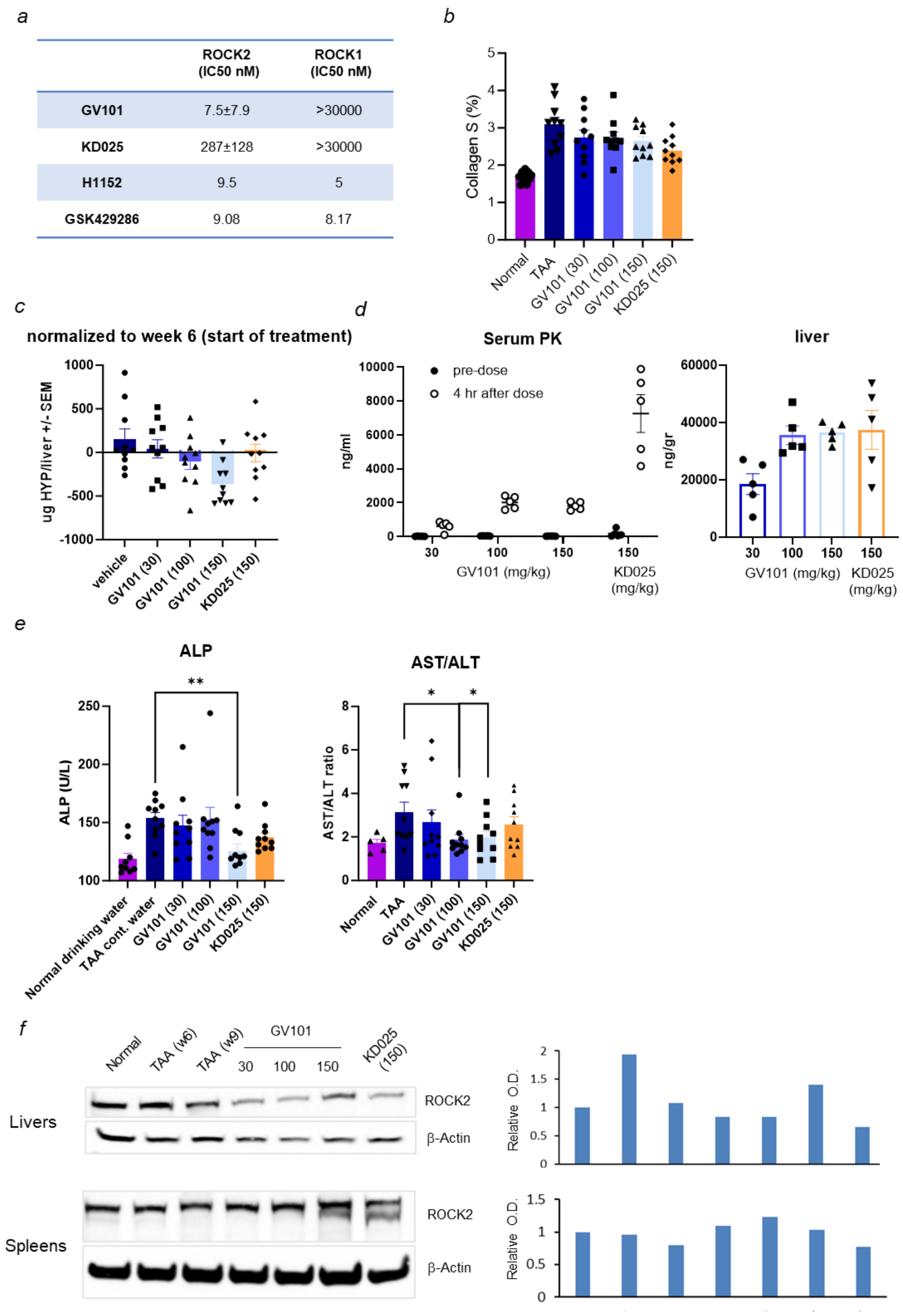

ROCK1 and ROCK2 IC50s were determined for ROCK inhibitors: GV101, KD025, H-1152 and GSK429286 by cell-free in vitro kinase assay (**a**). C57BL/6 female mice were treated with vehicle, GV101 (30, 100, and 150 mg/kg) or KD025 (150 mg/kg) by oral gavage on Week 6 after starting TAA in drinking water and continued for 3 weeks. Automatic quantification of picrosirius red (PSR)-stained liver section was performed by using imaging assay MorphoQuant®-NASH from digitalized images (**b**). Hydroxyproline measurements were normalized to Week 6 values when GV101 and KD025 treatment started (**c**). Drug levels were assessed in serum and liver tissues collected in week 9 (**d**). Liver enzyme analysis was performed on serum collected on Week 9 (**e**). Liver and spleen tissue lysates were prepared in RIPA buffer and levels of ROCK2 were determined by Western Blot (**f**). Western blots were quantified and normalized to the Normal control. Unpaired t-test statistical analysis was performed: * $p \leq 0.05$; ** $p \leq 0.01$.

Supplementary Figure 2. GV101 downregulates liver fibrosis induced by TAA in combination with Western Diet (WD) via targeting of inflammatory, fibrotic, and metabolic pathways.

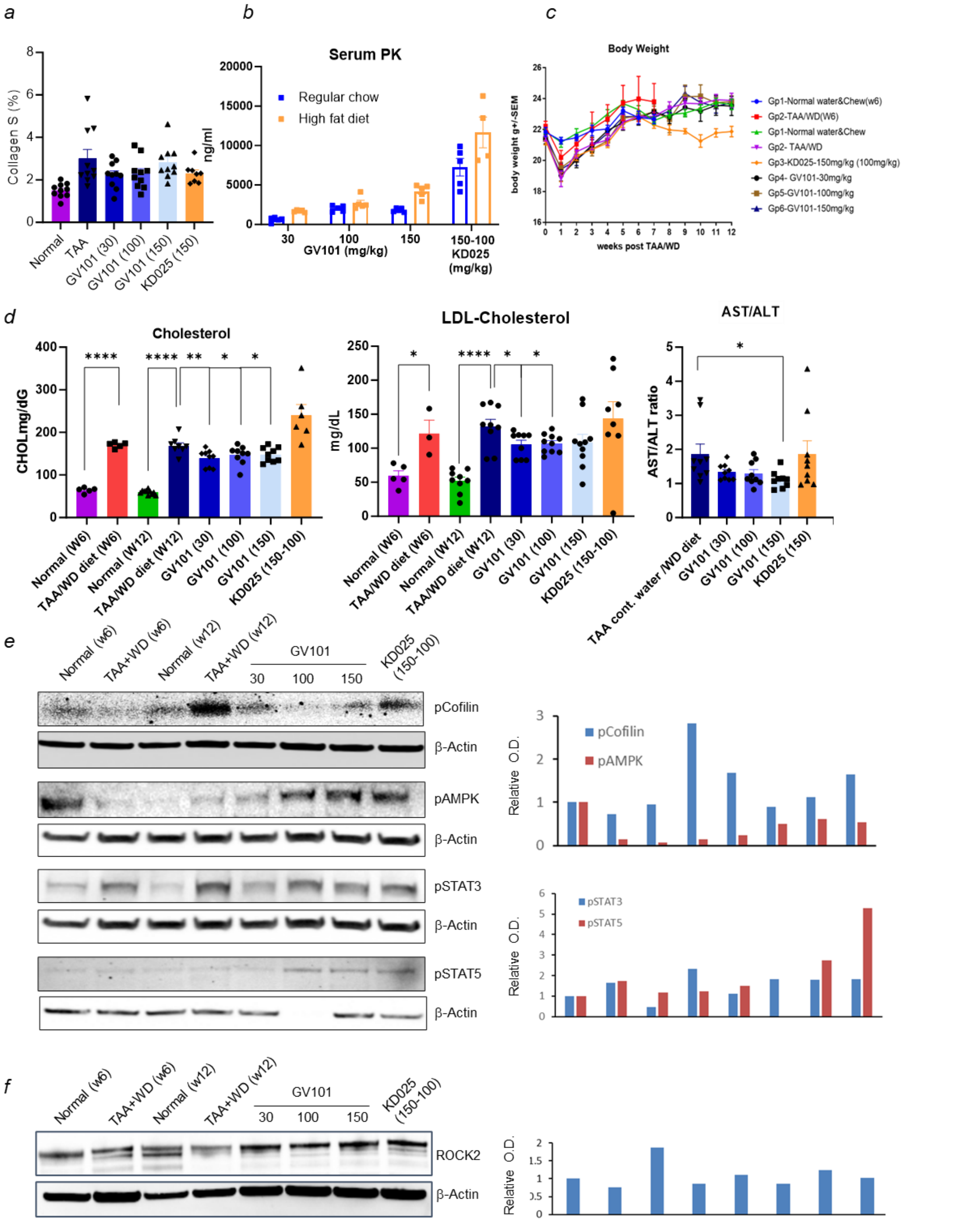

C57BL/6 female mice were treated with vehicle, GV101 (30, 100, and 150 mg/kg) or KD025 (150 mg/kg) by oral gavage on Week 6 after starting TAA in drinking water in combination with WD and continued for 6 weeks. KD025 dose was lowered to 100 mg/kg after 4 weeks of treatment due to toxicities. Automatic quantification of picrosirius red (PSR)-stained liver section was performed by using imaging assay MorphoQuant®-NASH from digitalized images (**a**). The levels of the inhibitors (**b**), total and LDL cholesterol and liver enzymes (**d**) were determined in serum collected in Week 12. Body weights were recorded twice weekly throughout the study period (**c**). Spleen (**e**) and liver (**d**) tissue lysates were prepared in RIPA buffer and levels of pCofilin, pAMPK, pSTAT3, pSTAT5 and ROCK2 were measured by Western Blot. Western blots were quantified and normalized to the Normal control. Unpaired t-test statistical analysis was performed: * $p \leq 0.05$; ** $p \leq 0.01$; *** $p \leq 0.001$; **** $p \leq 0.0001$.

Supplementary Figure 3. GV101 and KD25 inhibit adipogenesis of human subcutaneous preadipocytes and murine 3T3L1 cells and stimulate phosphorylation of AMPK.

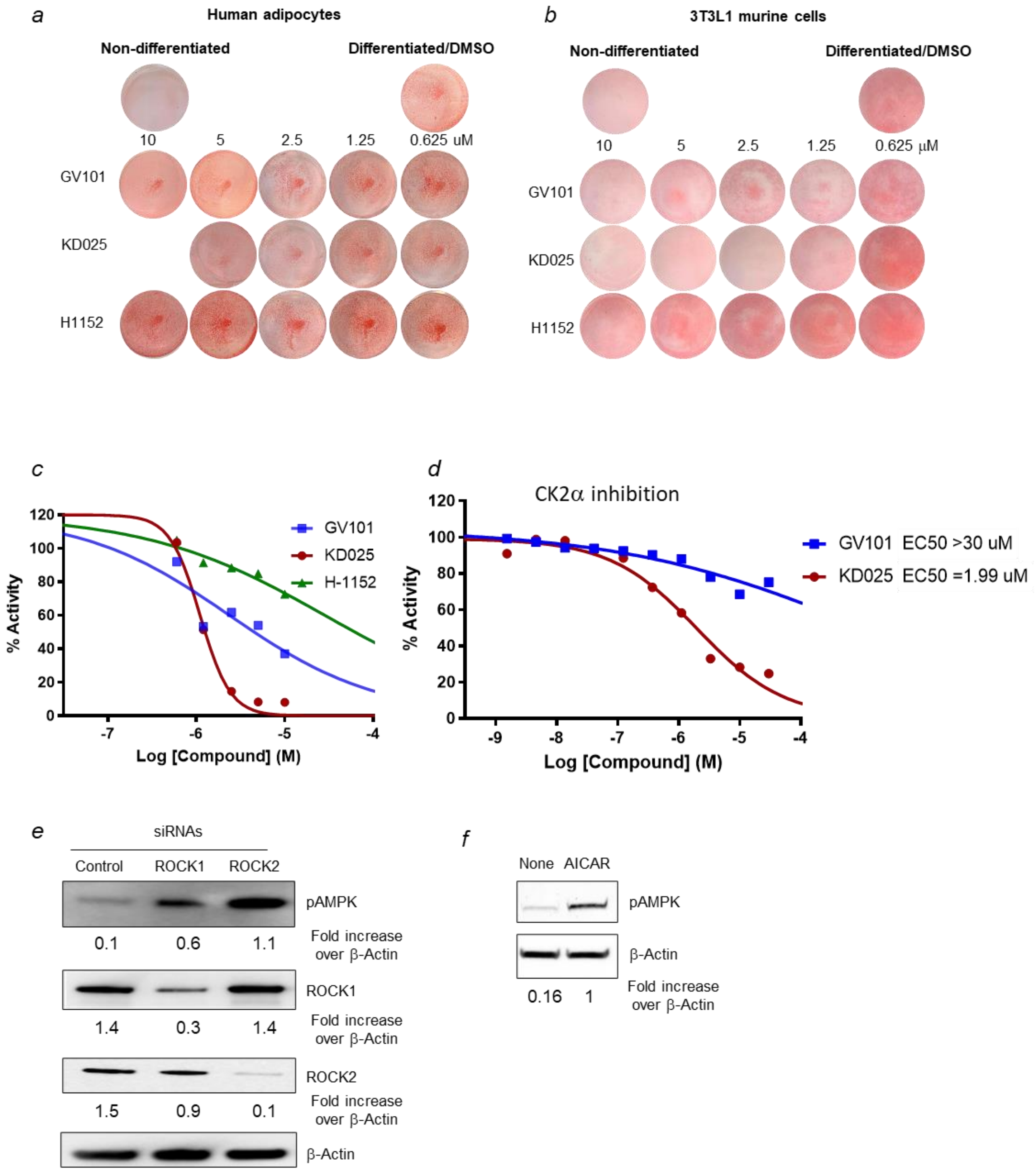

Human subcutaneous preadipocytes and murine 3T3L1 cells were induced adipogenesis with a differentiation cocktail medium (DM) for 7 days in the presence of indicated inhibitors. Cells were stained with Oil Red O on day 8 (**a** and **b**). The quantification of lipid accumulation was determined by measuring absorbance at 492 nM of extracted Oil Red O from stained cells, all the absorbance was normalized to vehicle (DMSO/DM) treated differentiated cells (**c**). Kinase profiling on CK2α was performed by Reaction Biology, compounds were tested in a 10-dose IC50 mode with a 3-fold serial dilution starting at 30 μM (**d**). Human subcutaneous preadipocytes were transfected with indicated siRNA by electroporation, whole cells extracts were obtained 48 hours after transfection and protein expression were analyzed by Western blot analysis (**e**). Human subcutaneous preadipocytes were treated with AICAR for 2 hours before whole cell extracts were collected for Western blot analysis of phosphorylated AMPK and β-actin (**f**). Western blots were quantified and normalized to the β-actin, and values are indicated under the corresponding immunoblots. The data (**a**, **b** and **c**) is representative of three repeated experiments.

Supplementary Figure 4. Selective ROCK2, but not pan-ROCK inhibitors potently down-regulate IL-17, IL-21 and CXCL13 secretion in human T cells.

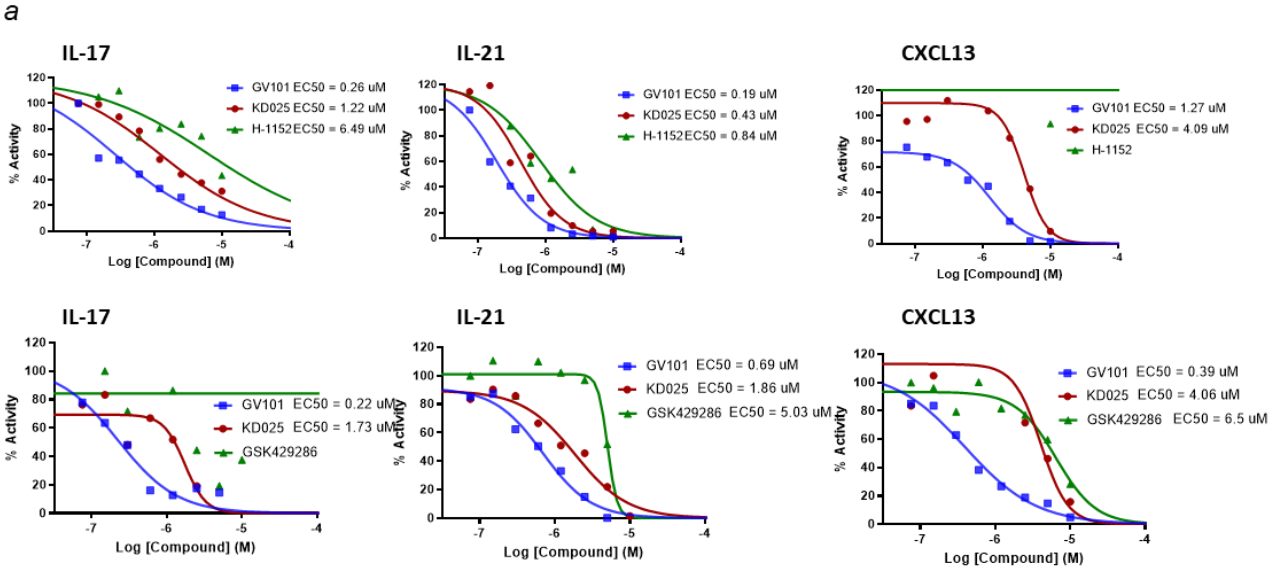

	IL-17 (IC50 μM)	IL-21 (IC50 μM)	CXCL13 (IC50 μM)
**GV101 (n=3)**	0.33±0.15	0.39±0.26	1.35±1
**KD025 (n=3)**	2.25±1.37	1.27±0.75	4.94±1.5

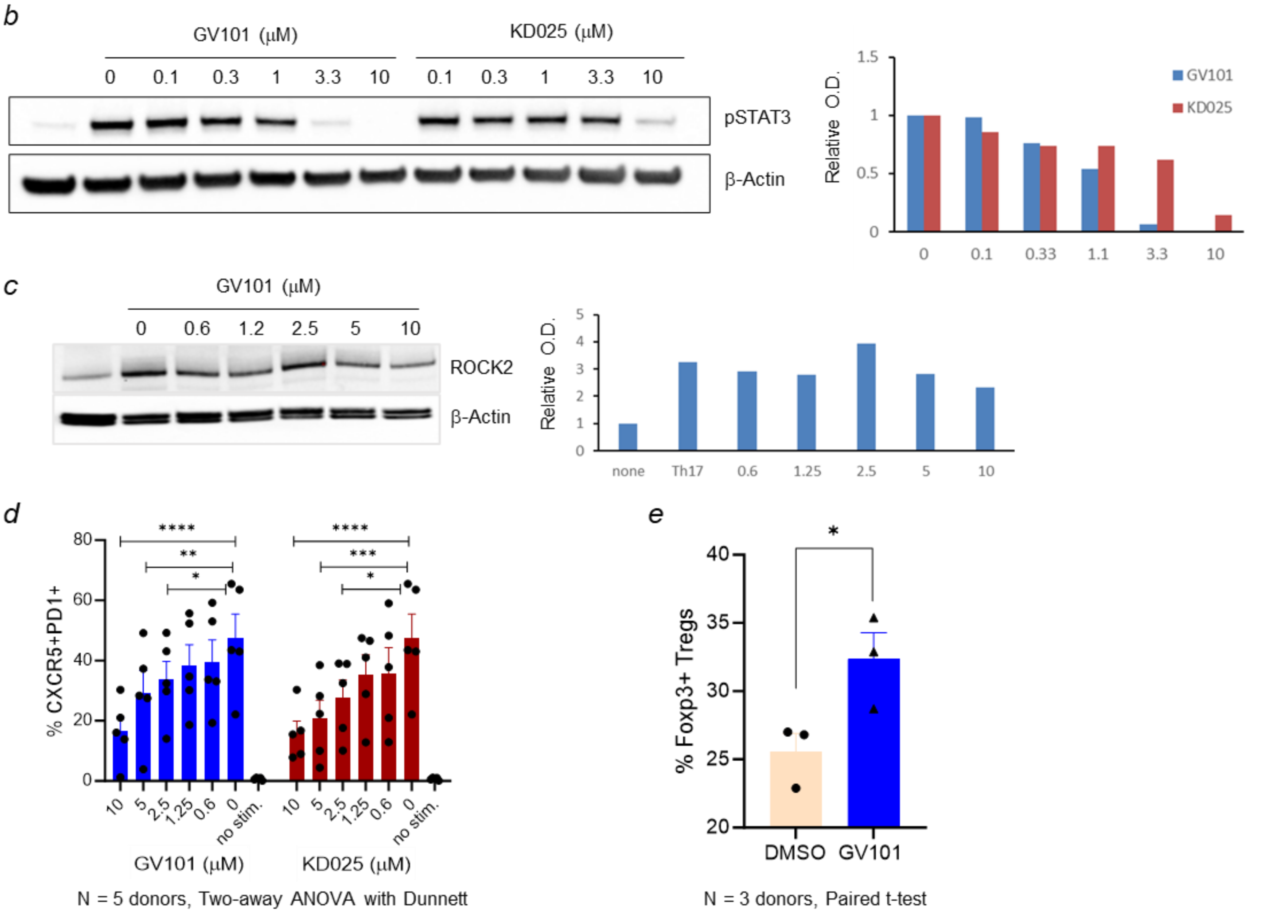

Peripheral blood human $CD4^+$ T cells were treated with indicated doses of selective ROCK2 inhibitors GV101 (**a**-**e**) and KD025 (**b**, **c** and **e**) or with pan ROCK inhibitors H-1152 and GSK429286 (**e**) for 2 hours and then stimulated by anti-CD3/28 mAbs in combination with IL-1β and TGF-β. IL-21, IL-17, and CXCL13 secretion was analyzed by ELISA after 48 h. Whole cellular extracts were prepared and analyzed by Western blot (**b, c**). Western blots were quantified and normalized to the DMSO control. The percentages of $CXCR5^+PD1^+$ cells (**d**) and $Foxp3^+$ cells (**e**) were determined by flow cytometric analysis. Two-away ANOVA (with Dunnett correction) statistical analysis was performed: * $p \leq 0.05$; ** $p \leq 0.01$; *** $p \leq 0.001$; **** $p \leq 0.0001$ (**d**). Paired t-test statistical analysis was performed: * $p \leq 0.05$ (**e**).

Supplementary Figure 5. GV101 inhibits the pro-inflammatory response of primary PBMCs, Monocytes, MDM and Kupffer Cells in mice and human.

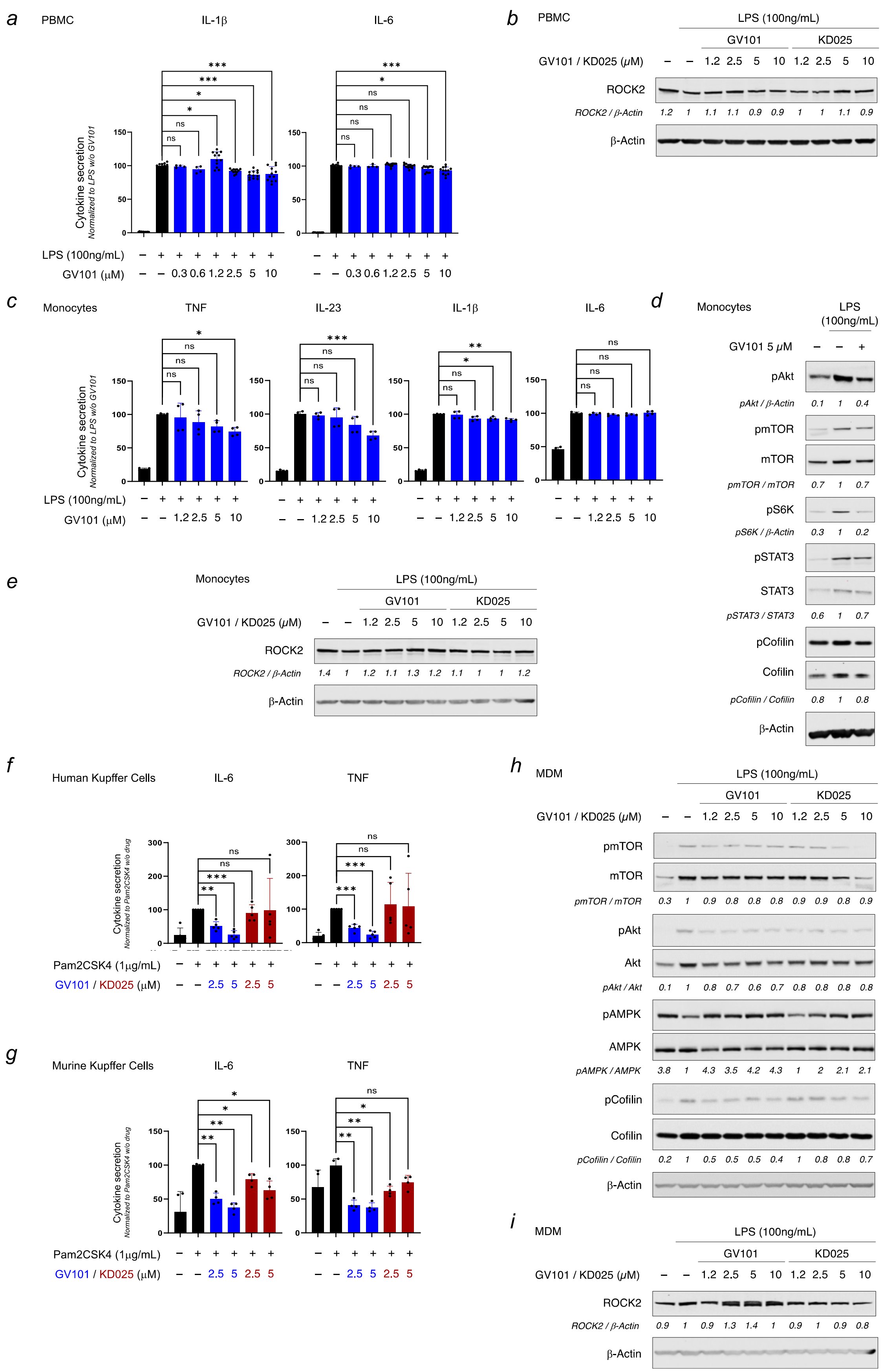

PBMCs were treated with indicated doses of GV101 1.5 hr before stimulation with LPS. After 24 hrs, IL-1β and IL-6 secretion in supernatants was analyzed by ELISA and normalized (***a***, n=12 except GV101 0.3 and 0.6uM: n=4) and Western blots for the indicated proteins were performed on whole cell extracts (***b***). Human primary monocytes were treated with indicated doses of GV101 1.5 hr before stimulation with LPS. After 24 hrs, TNF, IL-23, IL-1β and IL-6 secretion in supernatants was analyzed by ELISA and normalized (***b***, n=4) and Western blots for the indicated proteins were performed on whole cell extracts (***d, e***). Human (***f***) or murine (***g***) primary Kupffer cells were treated with indicated doses of GV101 or KD025 1.5 hr before stimulation with Pam2CSK4 and 24 hrs before collection of Kupffer cells supernatants for analysis of IL-6 and TNF secretion by ELISA followed by normalization (***f***, n=5; ***g***, n=4). MDM were treated with indicated doses of GV101 or KD025 1.5 hr before stimulation with LPS. After 24 hrs, Western blots for the indicated proteins were performed on whole cell extracts (***h, i***). All experiments represent at least 4 independent repeats. Graphs represent mean +/- s.e.m. In (***a***), (***c***), (***f***) and (***g***), one way ANOVA was performed followed by multiple comparisons Sidak tests to allow two-by-two comparisons. ns: not significant, * $p \leq 0.05$; ** $p \leq 0.01$; *** $p \leq 0.001$. In (***b***), (***d***), (***e***), (***h***) and (***i***), Western blots were quantified and normalized to the LPS-stimulated conditions, and values are indicated under the corresponding immunoblots.
