# Supplementary material for: Selectivity matters: selective ROCK2 inhibitor ameliorates established liver fibrosis via targeting inflammation, fibrosis, and metabolism
Source: Commun Biol. 2023 Nov 18;6:1176. doi: 10.1038/s42003-023-05552-0 (PMC10657369; doi:10.1038/s42003-023-05552-0)
Supplement: Supplementary file 3 — Description of Additional Supplementary Data [file 42003_2023_5552_MOESM3_ESM.docx]

**Description of Additional Supplementary Files**

**File name:** Supplementary Data 1

**Description:** the raw/source data behind graphs in the main figures

**File name:** Supplementary Data 2

**Description:** the raw/source data behind the graphs in the supplementary figures

**File name:** Supplementary Data 3

**Description:** the unprocessed Western Blot scans for images included in the main and supplementary figures
