# Supplementary material for: Selectivity matters: selective ROCK2 inhibitor ameliorates established liver fibrosis via targeting inflammation, fibrosis, and metabolism
Source: Commun Biol. 2023 Nov 18;6:1176. doi: 10.1038/s42003-023-05552-0 (PMC10657369; doi:10.1038/s42003-023-05552-0)

Figure 1d

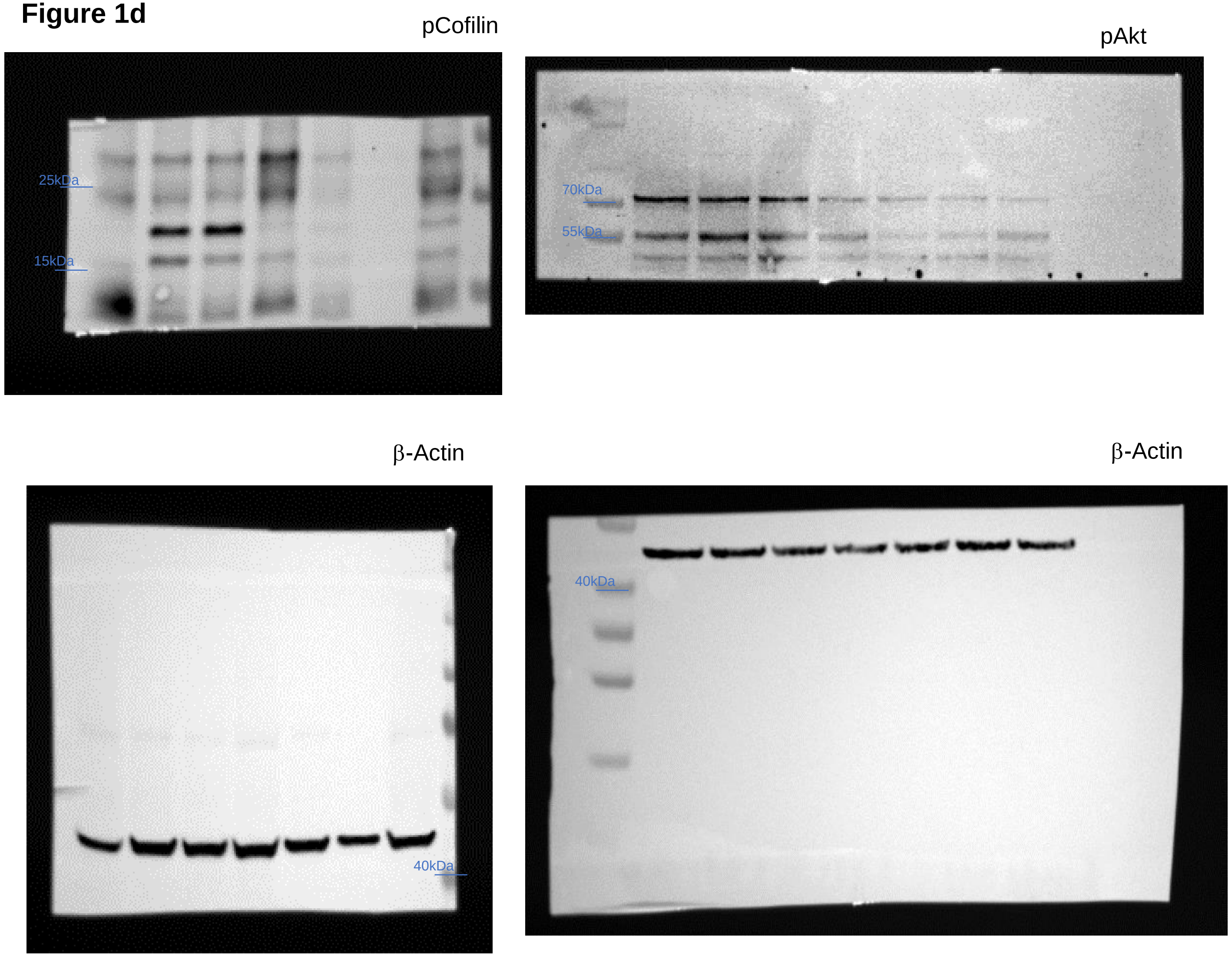

Figure 1e

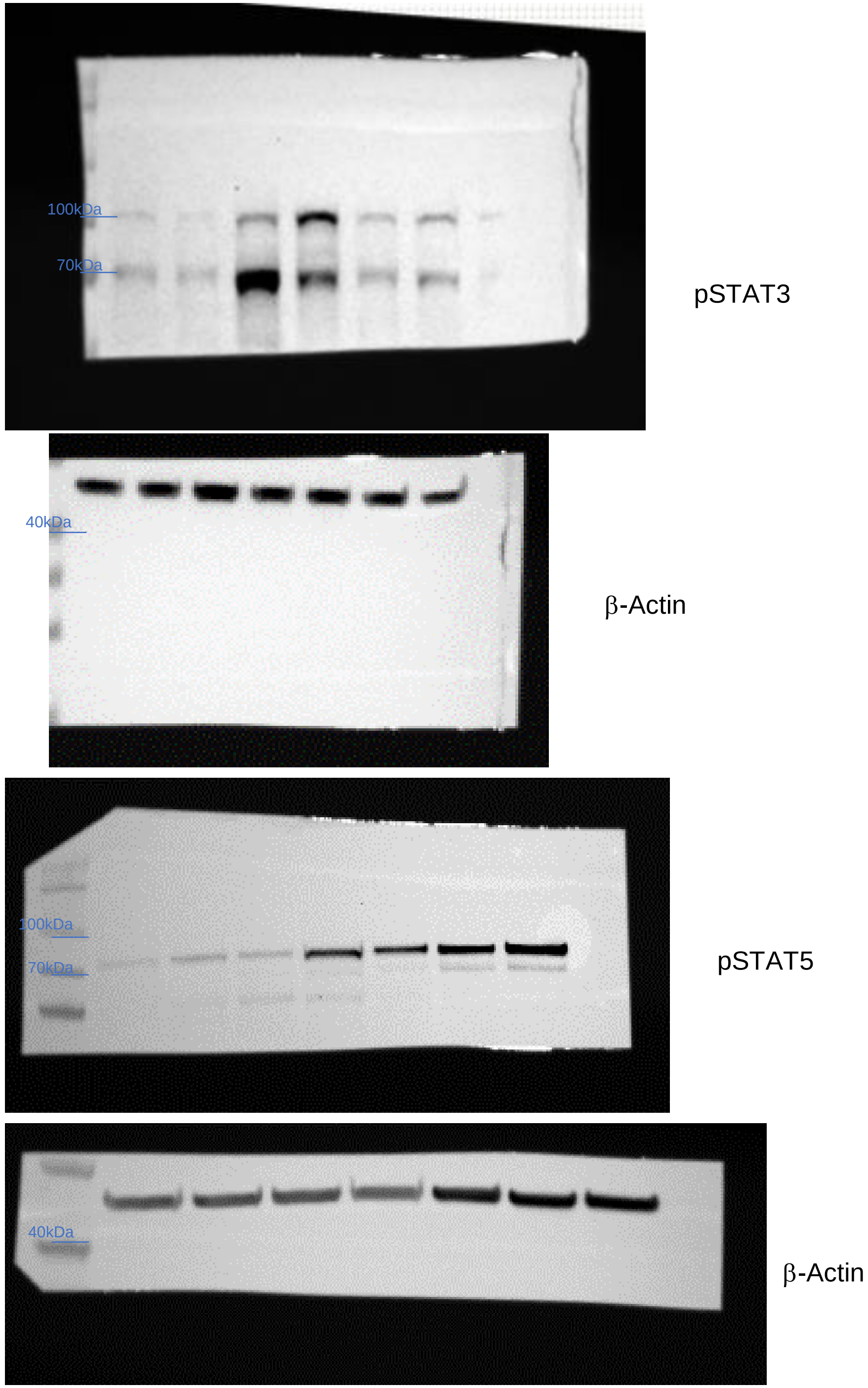

Figure 2e

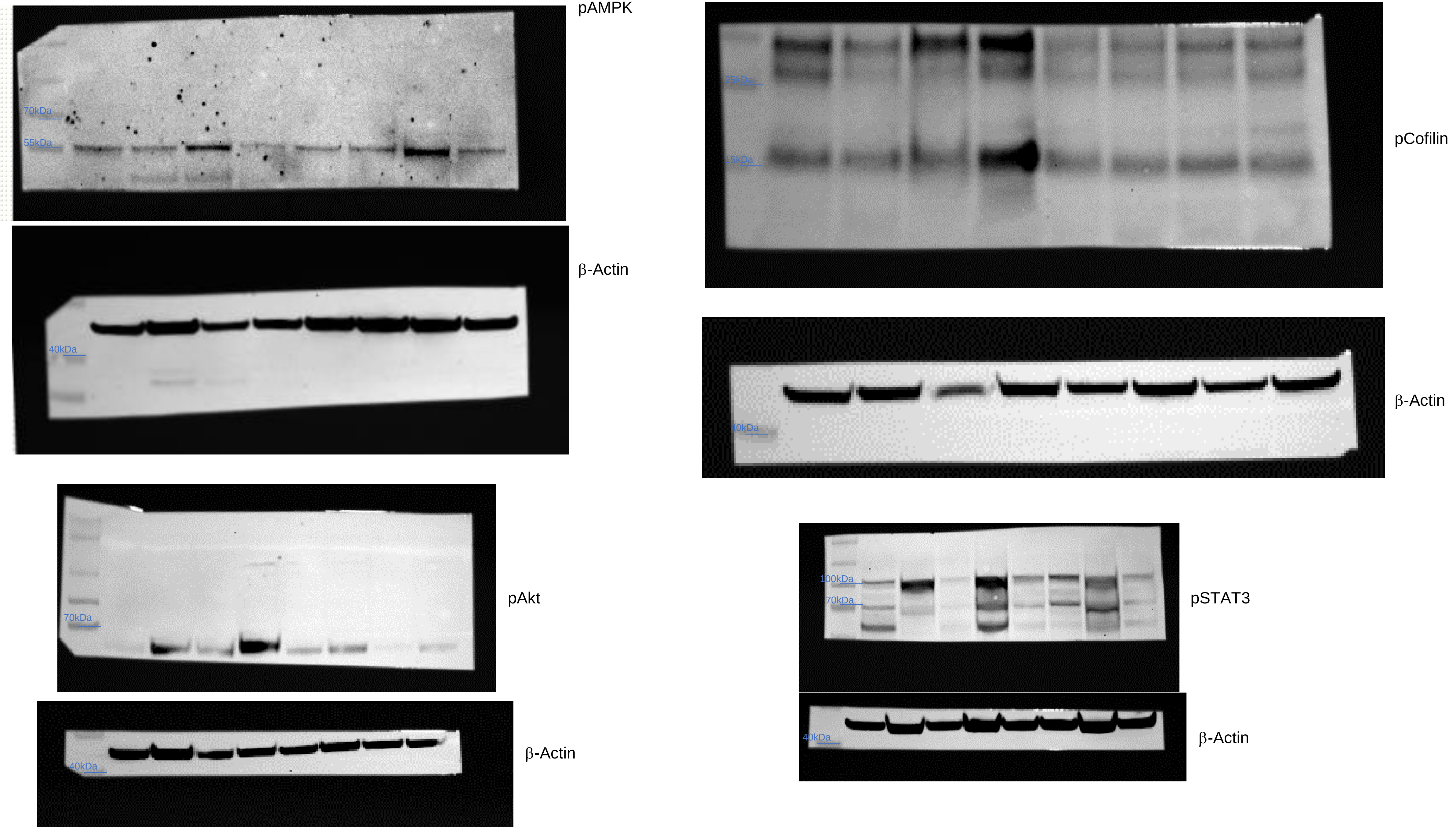
pAMPK
70kDa
55kDa
β-Actin
40kDa
pCofilin
25kDa
15kDa
β-Actin
40kDa
pAkt
70kDa
β-Actin
40kDa
pSTAT3
100kDa
70kDa
β-Actin
40kDa

Figure 3c

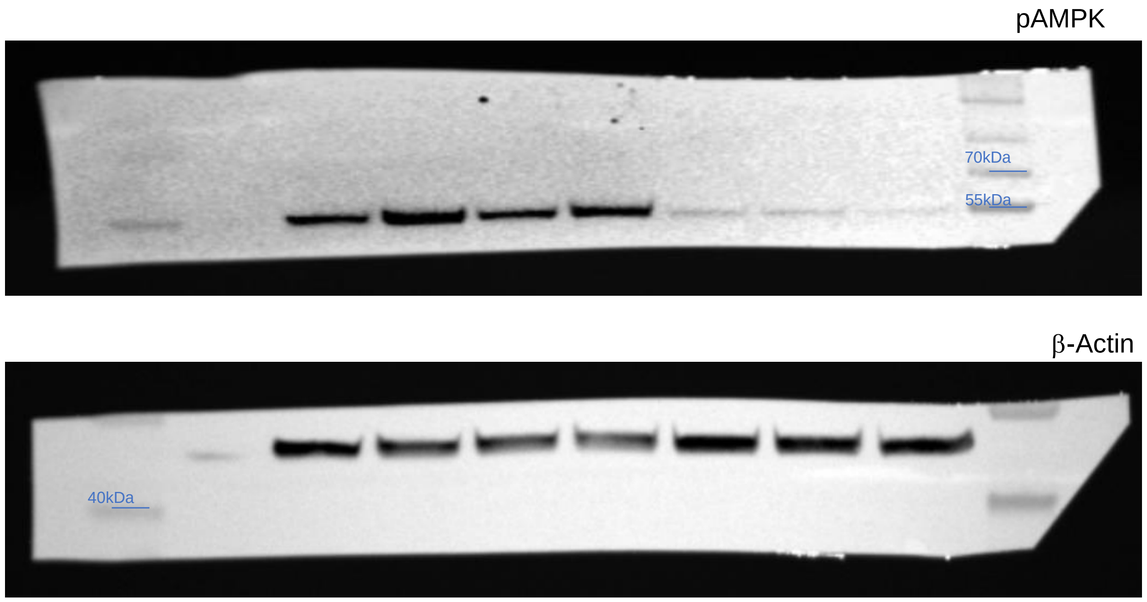

Figure 3d

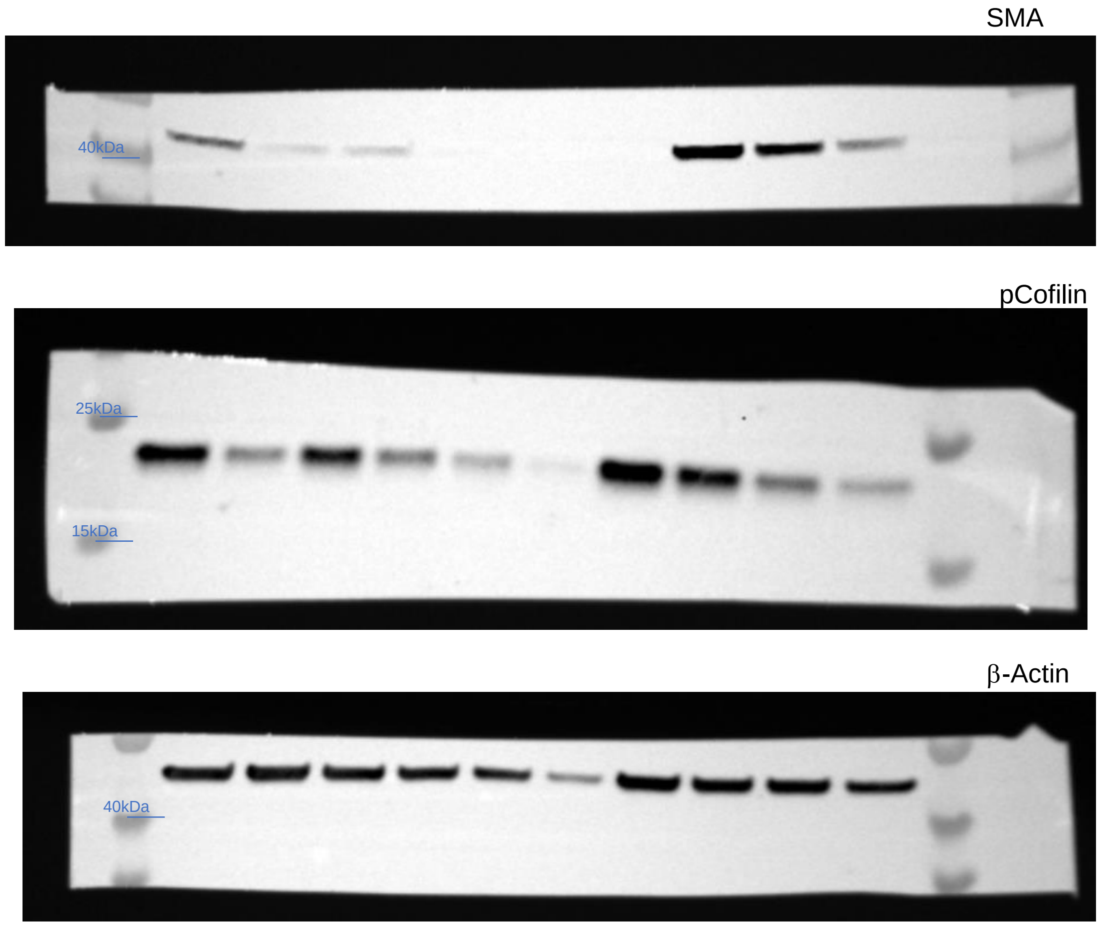

Figure 4b

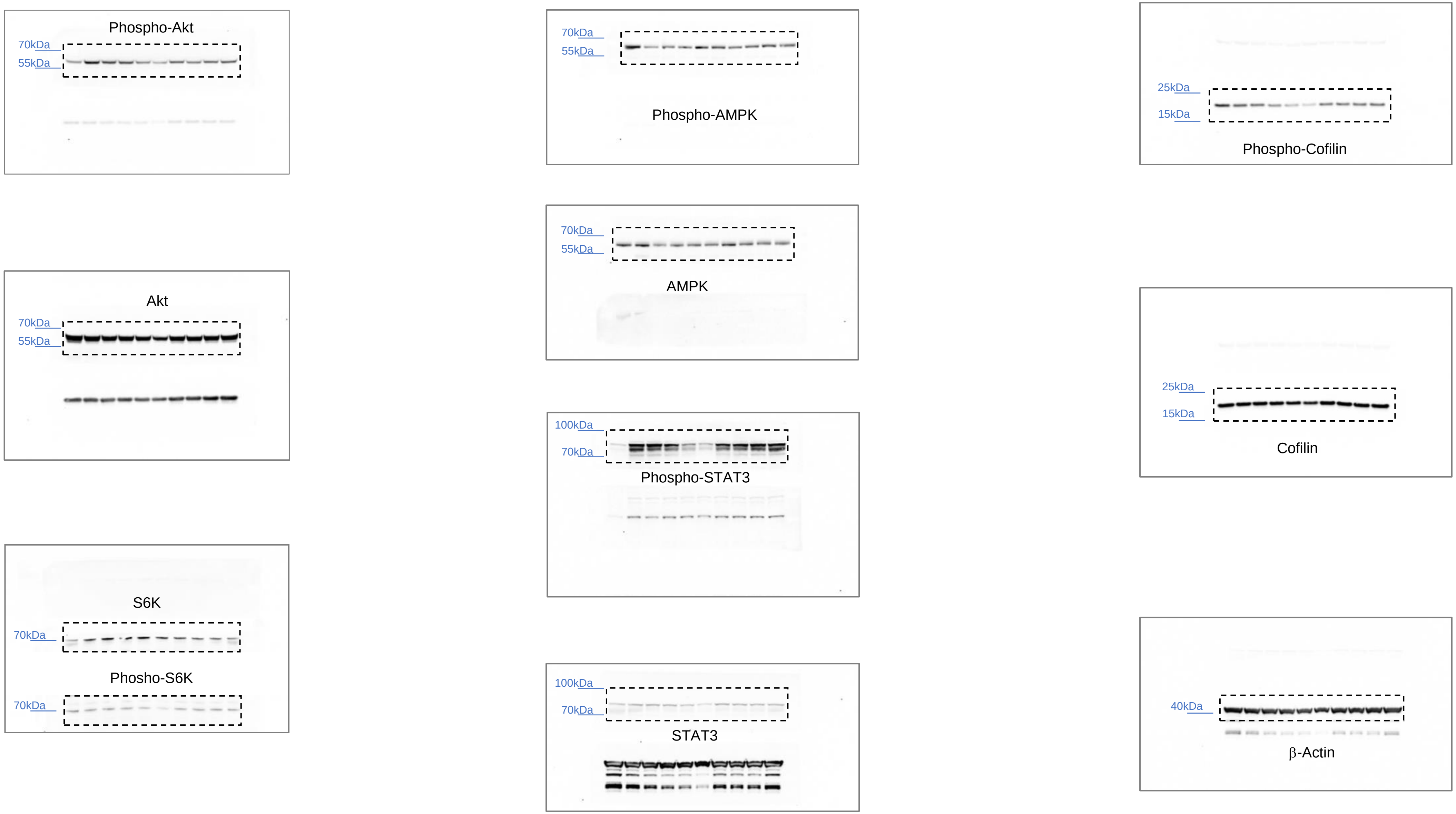

Phospho-Akt
70kDa
55kDa
Akt
70kDa
55kDa
S6K
70kDa
Phosho-S6K
70kDa
70kDa
55kDa
Phospho-AMPK
70kDa
55kDa
AMPK
100kDa
70kDa
Phospho-STAT3
100kDa
70kDa
STAT3
25kDa
15kDa
Phospho-Cofilin
25kDa
15kDa
Cofilin
40kDa
β-Actin

Figure 4e

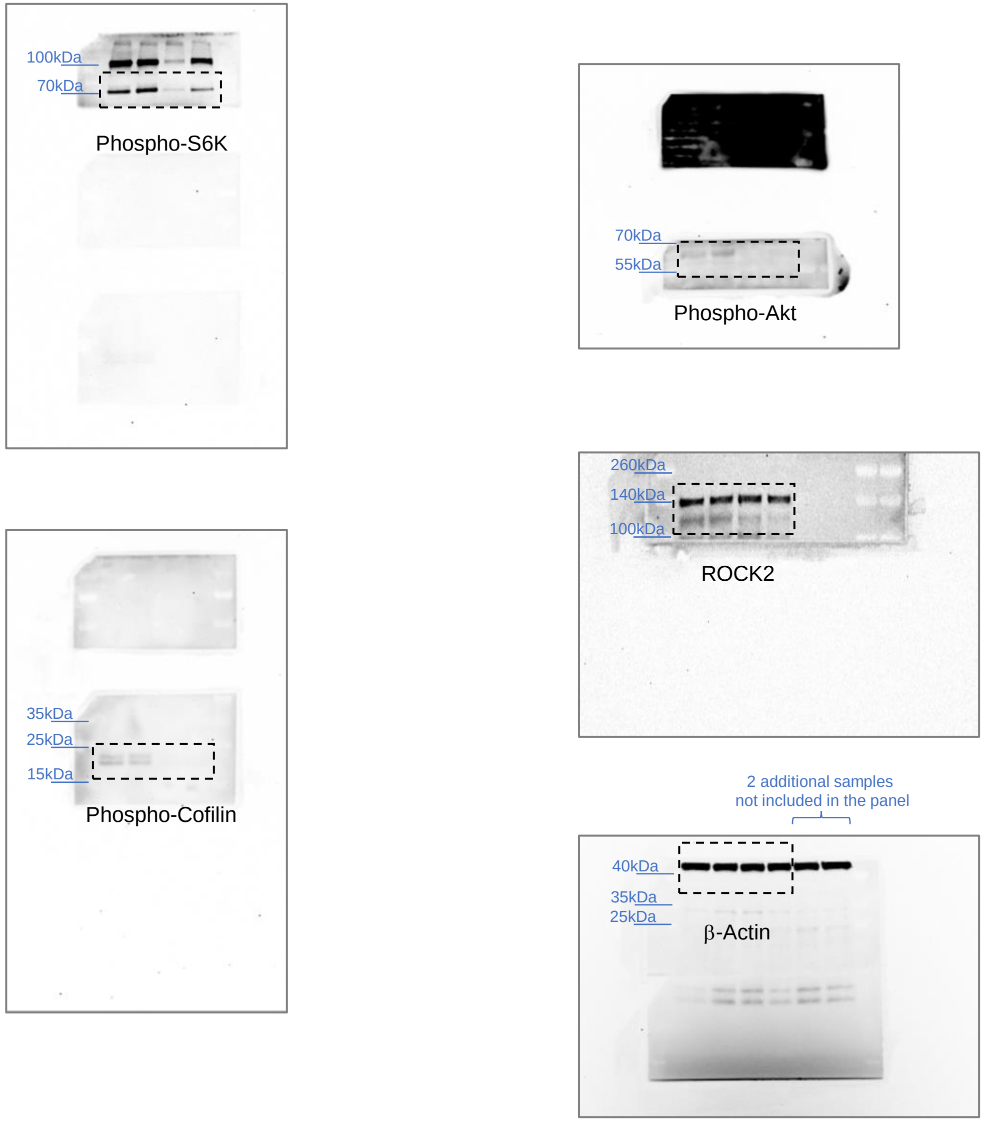
100kDa
70kDa
Phospho-S6K
70kDa
55kDa
Phospho-Akt
260kDa
140kDa
100kDa
ROCK2
35kDa
25kDa
15kDa
Phospho-Cofilin
2 additional samples
not included in the panel
40kDa
35kDa
25kDa
β-Actin

Supplementary Figure 1f

Livers

ROCK2

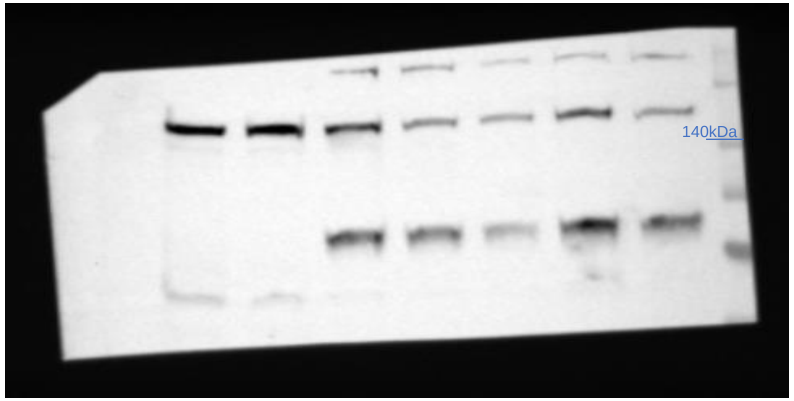

β-Actin

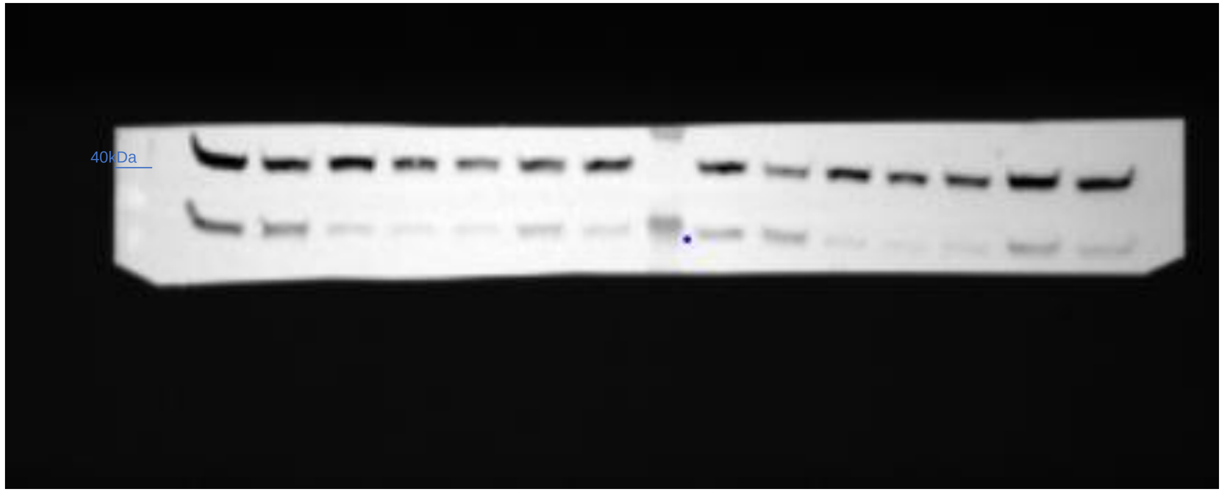

Spleens

ROCK2

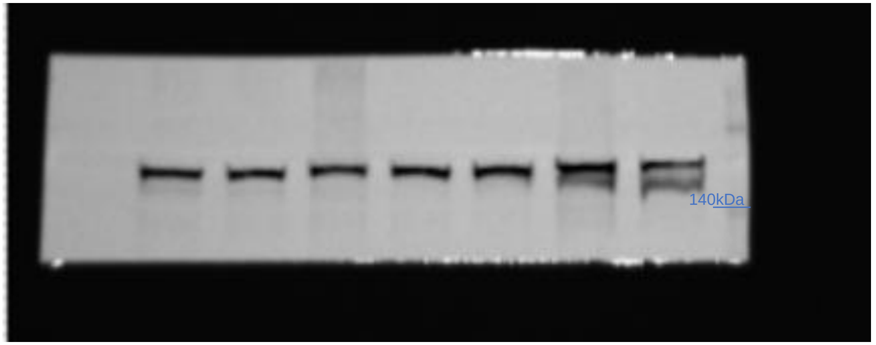

β-Actin

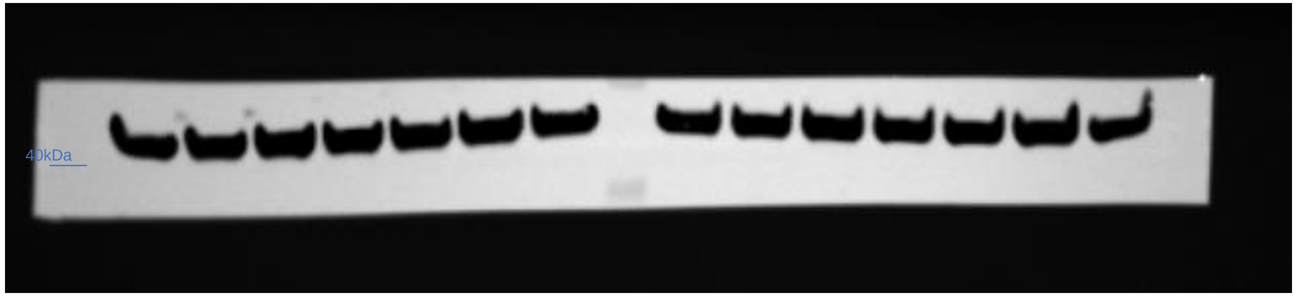

Supplementary Figure 2e

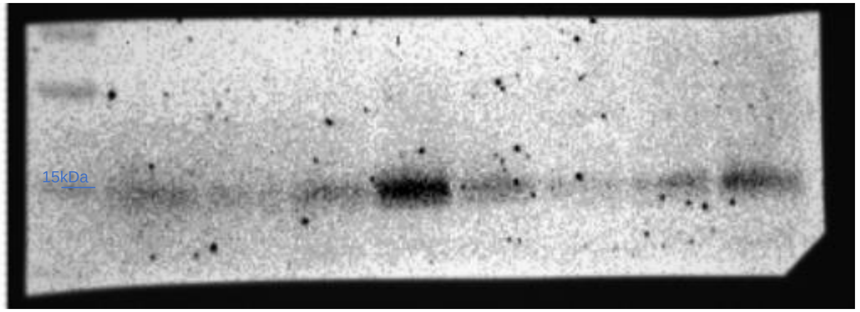

pCofilin

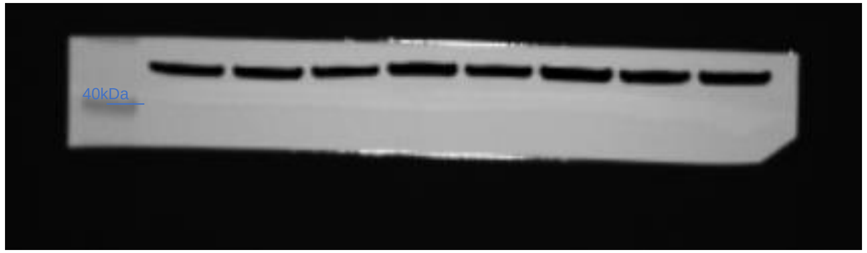

β-Actin

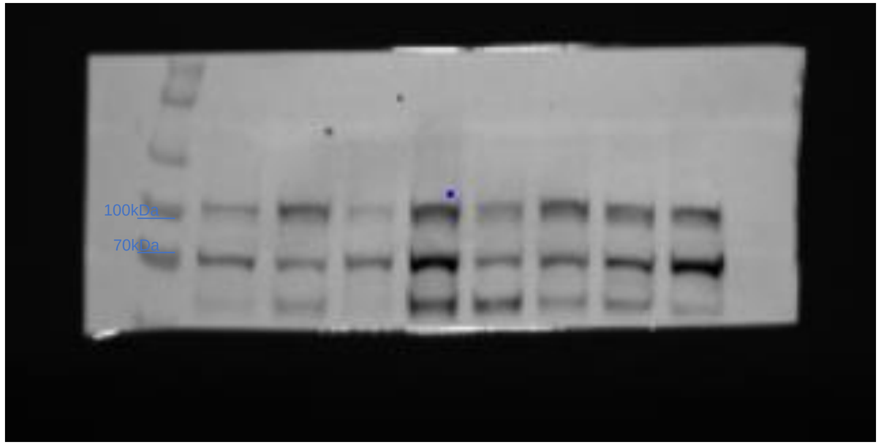

pSTAT3

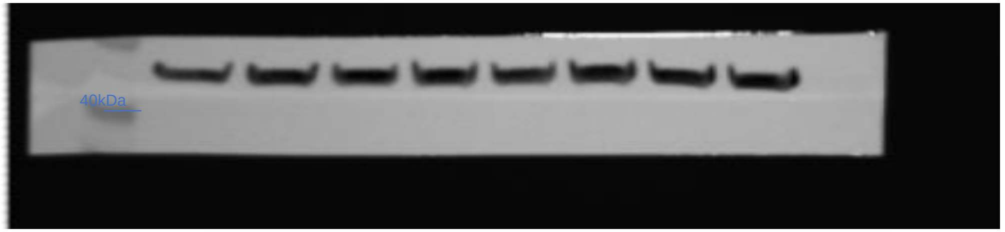

β-Actin

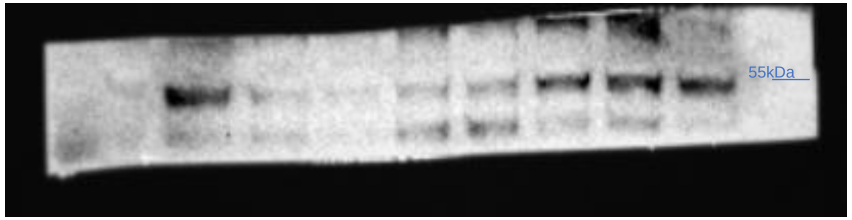

pAMPK

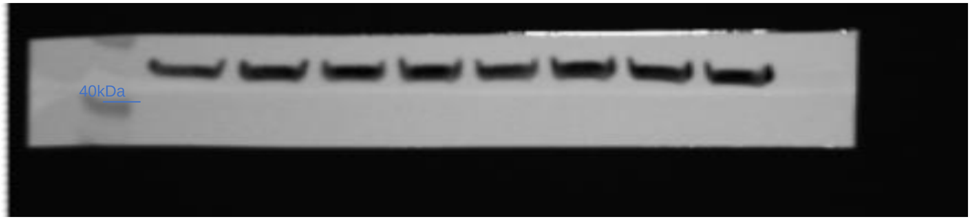

β-Actin

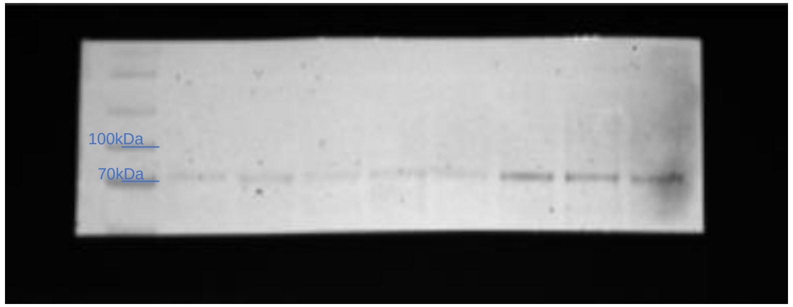

pSTAT5

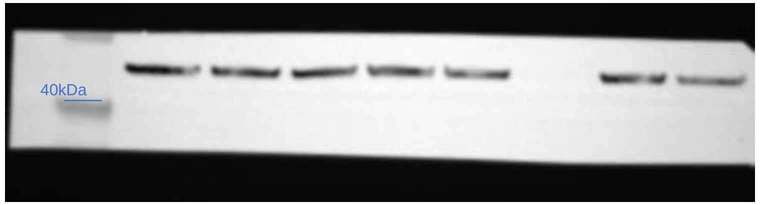

β-Actin

Supplementary Figure 2f

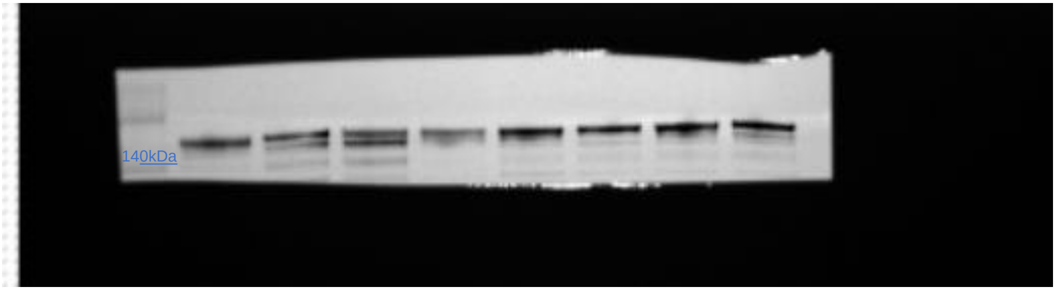

ROCK2

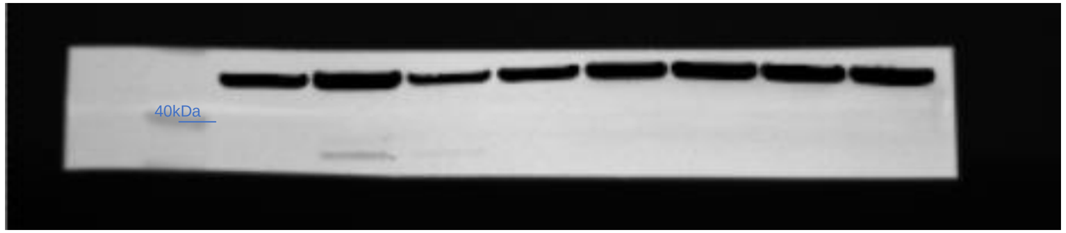

β-Actin

Supplementary Figure 3e

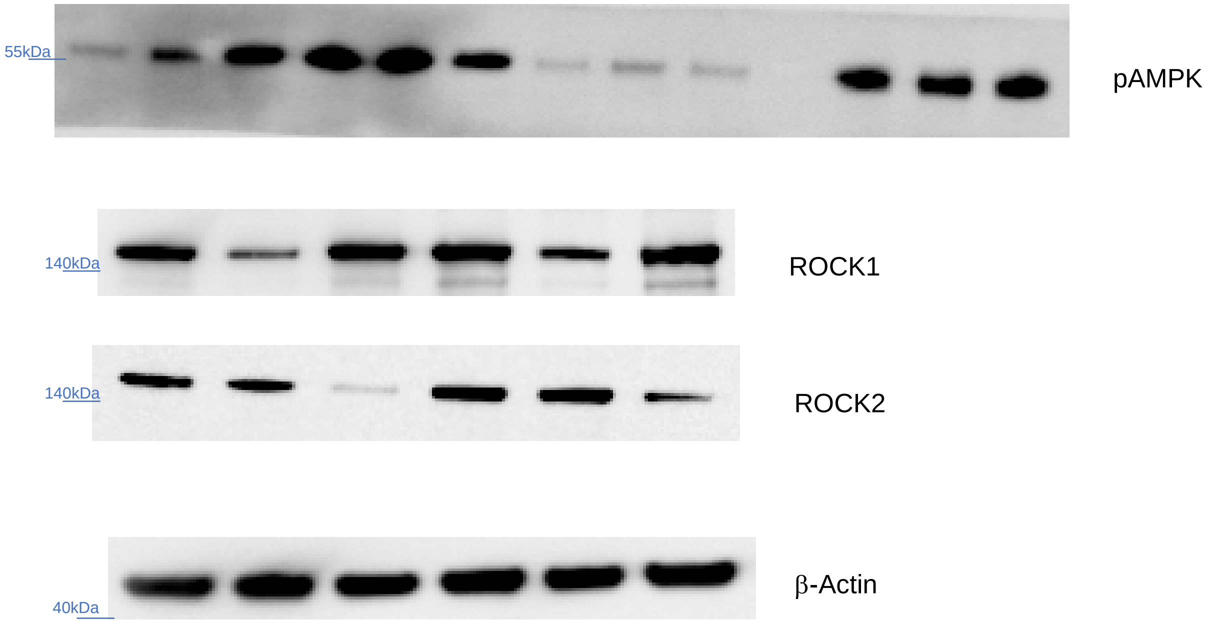

Supplementary Figure 3f

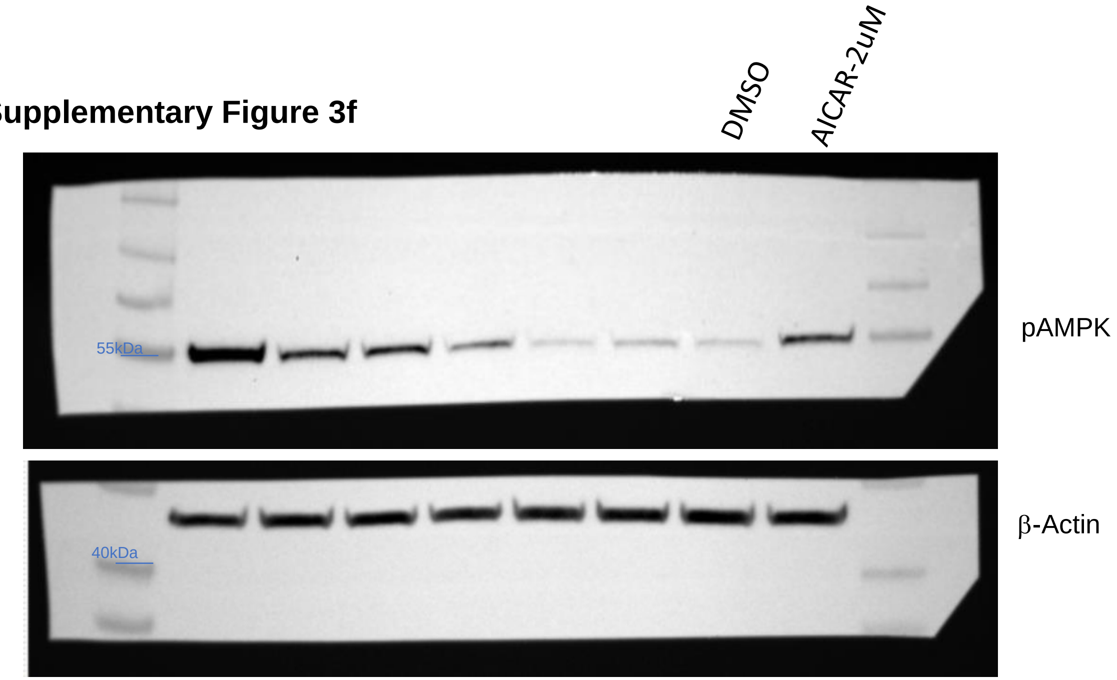

Supplementary Figure 4b

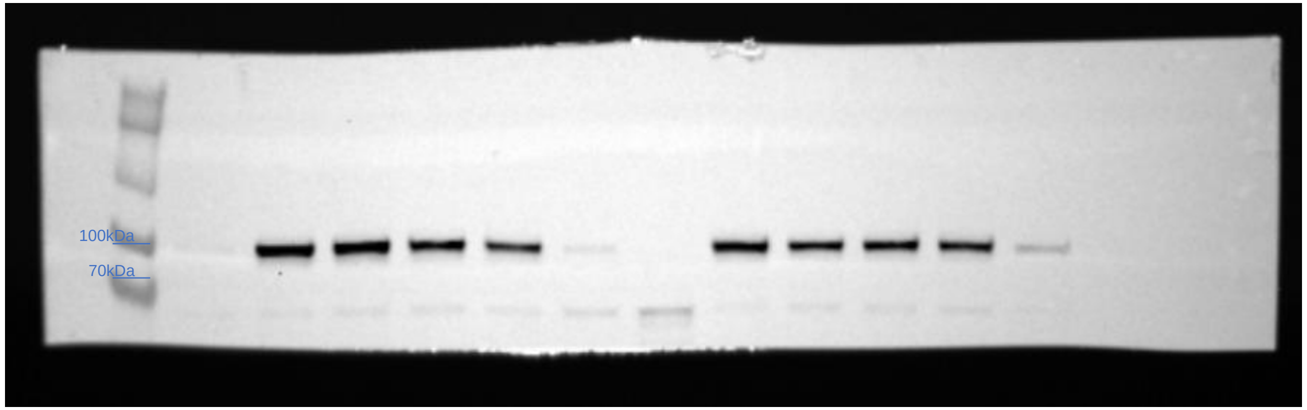

pSTAT3

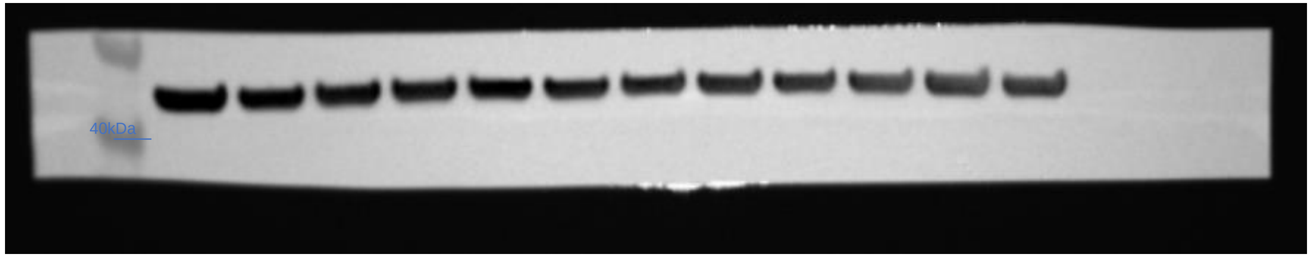

β-Actin

Supplementary Figure 4c

ROCK2

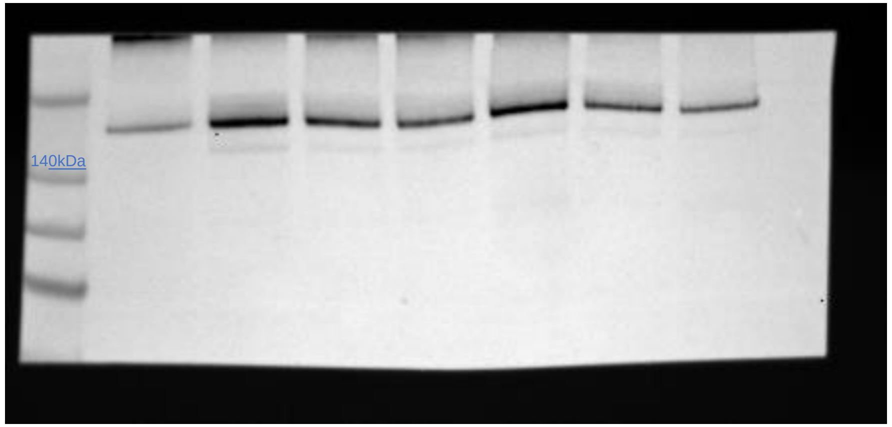

β-Actin

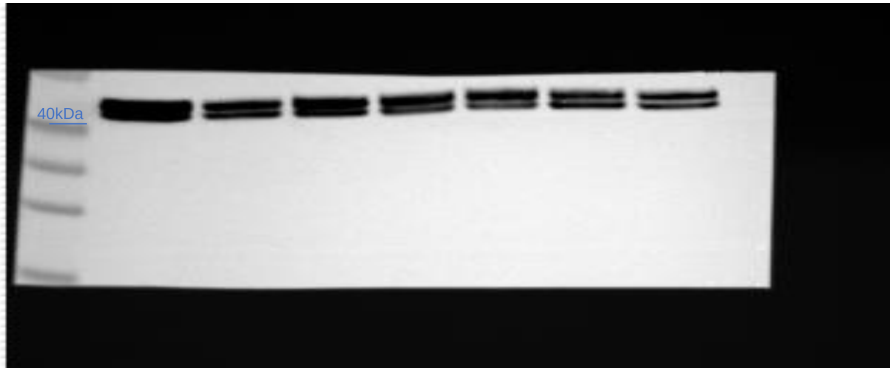

Supplementary Figure 5b

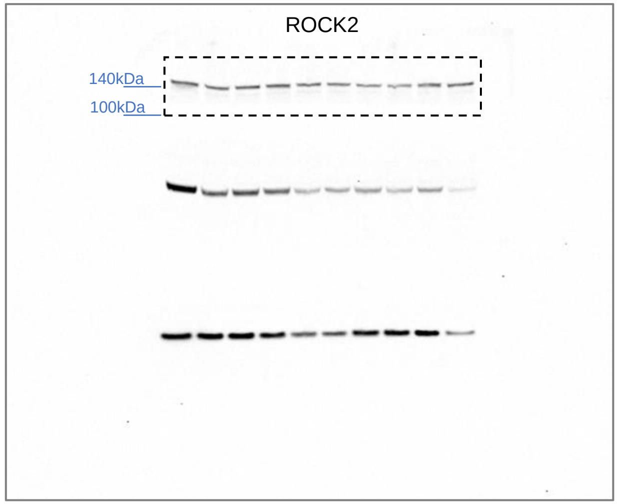

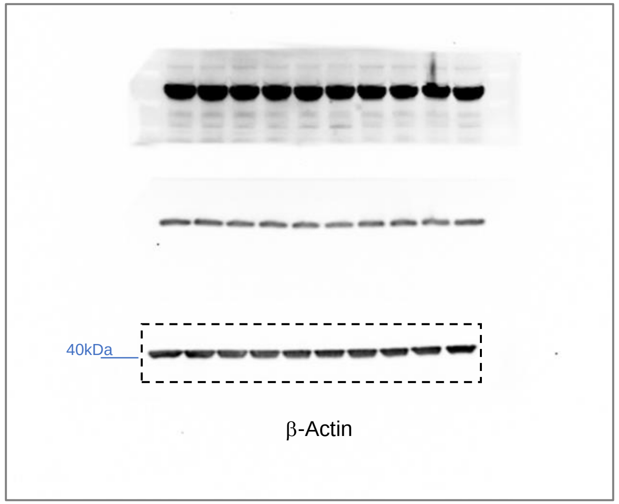

Supplementary Figure 5d

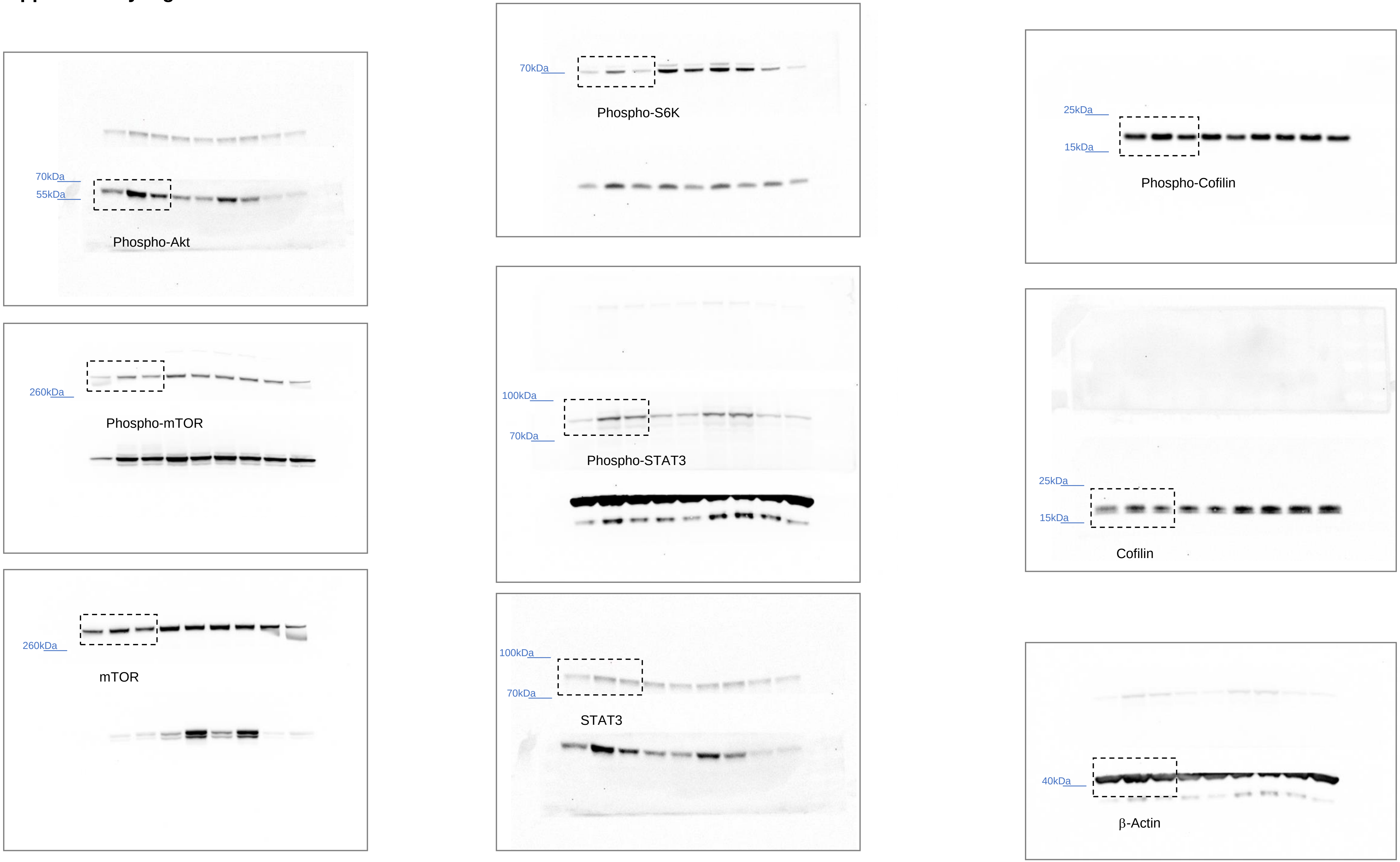

Supplementary Figure 5e

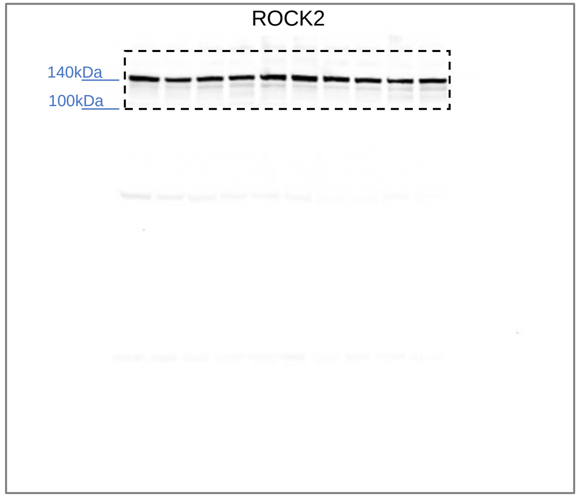

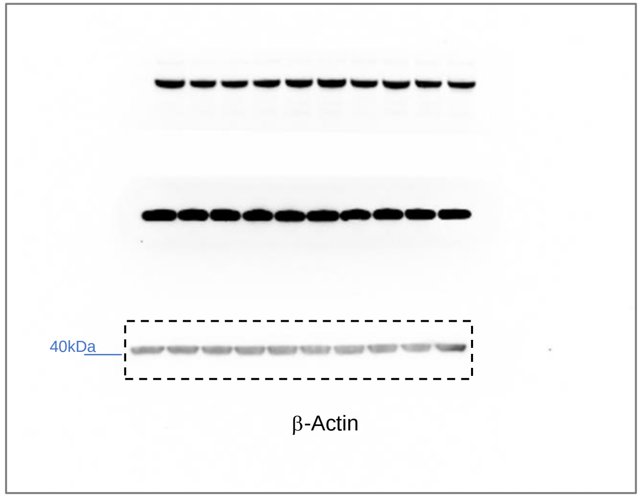

Supplementary Figure 5h

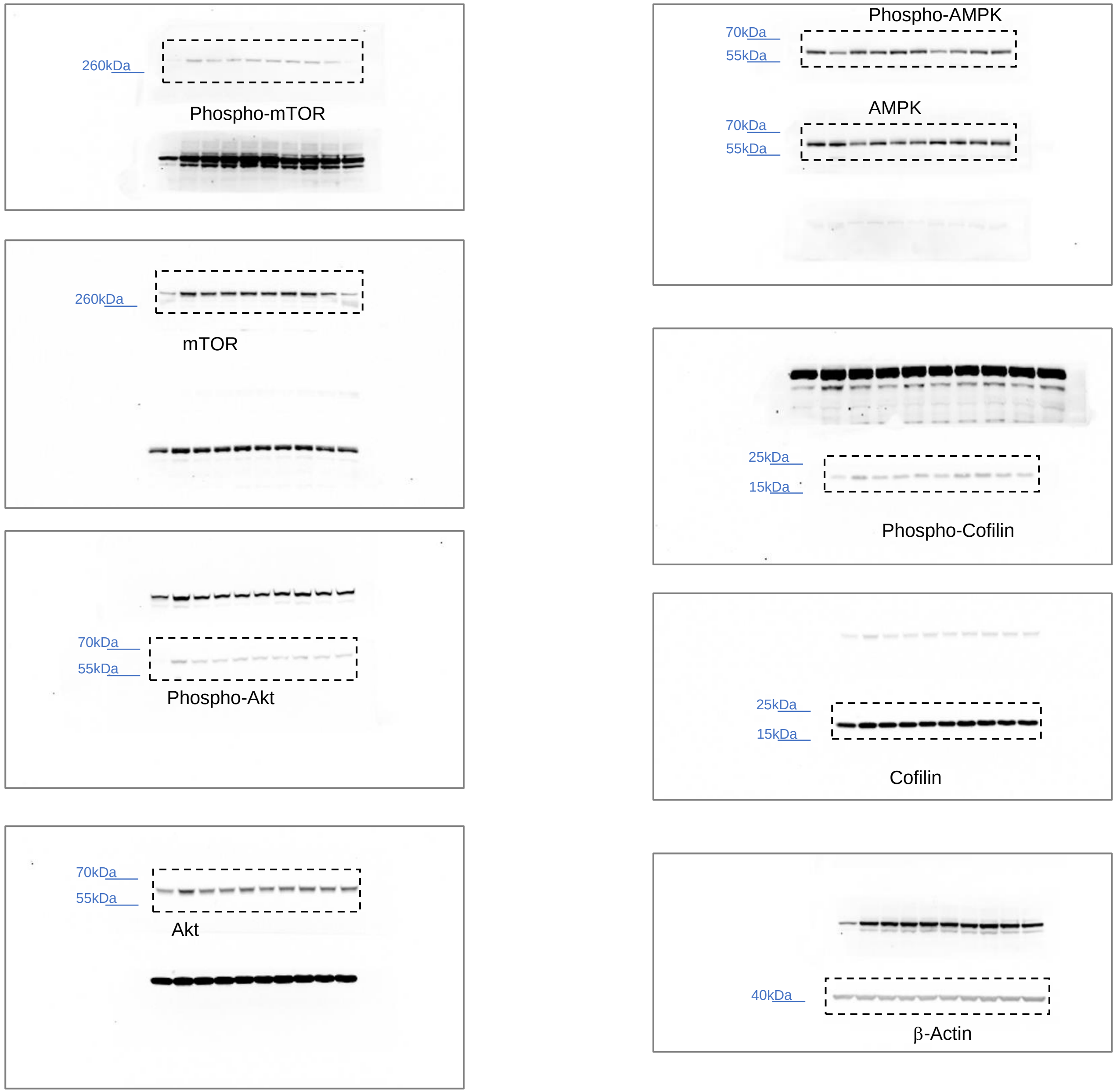

Supplementary Figure 5i

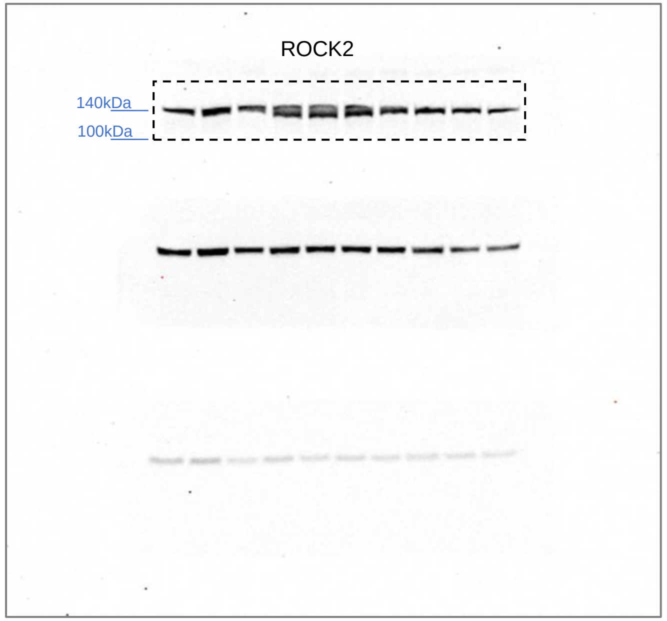

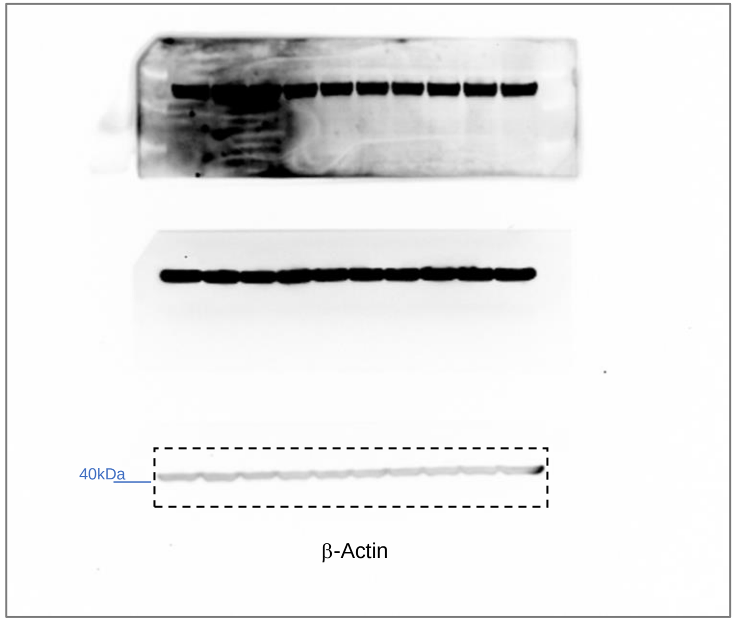

Supplement: Supplementary file 6 — Supplementary data 3 [file 42003_2023_5552_MOESM6_ESM.pdf]
