# Supplementary material for: Selectivity matters: selective ROCK2 inhibitor ameliorates established liver fibrosis via targeting inflammation, fibrosis, and metabolism
Source: Commun Biol. 2023 Nov 18;6:1176. doi: 10.1038/s42003-023-05552-0 (PMC10657369; doi:10.1038/s42003-023-05552-0)
Supplement: Supplementary file 7 — Reporting Summary [file 42003_2023_5552_MOESM7_ESM.pdf]

nature portfolio

Corresponding author(s): Alexandra Zanin-Zhorov

Last updated by author(s): Nov 3, 2023

Reporting Summary

Nature Portfolio wishes to improve the reproducibility of the work that we publish. This form provides structure for consistency and transparency in reporting. For further information on Nature Portfolio policies, see our Editorial Policies and the Editorial Policy Checklist.

Statistics

For all statistical analyses, confirm that the following items are present in the figure legend, table legend, main text, or Methods section.

n/a	Confirmed	
☐	☒	The exact sample size (*n*) for each experimental group/condition, given as a discrete number and unit of measurement
☐	☒	A statement on whether measurements were taken from distinct samples or whether the same sample was measured repeatedly
☐	☒	The statistical test(s) used AND whether they are one- or two-sided<br>*Only common tests should be described solely by name; describe more complex techniques in the Methods section.*
☒	☐	A description of all covariates tested
☐	☒	A description of any assumptions or corrections, such as tests of normality and adjustment for multiple comparisons
☐	☒	A full description of the statistical parameters including central tendency (e.g. means) or other basic estimates (e.g. regression coefficient) AND variation (e.g. standard deviation) or associated estimates of uncertainty (e.g. confidence intervals)
☐	☒	For null hypothesis testing, the test statistic (e.g. F, t, r) with confidence intervals, effect sizes, degrees of freedom and P value noted<br>*Give P values as exact values whenever suitable.*
☒	☐	For Bayesian analysis, information on the choice of priors and Markov chain Monte Carlo settings
☒	☐	For hierarchical and complex designs, identification of the appropriate level for tests and full reporting of outcomes
☒	☐	Estimates of effect sizes (e.g. Cohen's *d*, Pearson's *r*), indicating how they were calculated

Our web collection on statistics for biologists contains articles on many of the points above.

Software and code

Policy information about availability of computer code

Data collection	N/A
Data analysis	Data analysis was performed using GraphPad / PRISM.

For manuscripts utilizing custom algorithms or software that are central to the research but not yet described in published literature, software must be made available to editors and reviewers. We strongly encourage code deposition in a community repository (e.g. GitHub). See the Nature Portfolio guidelines for submitting code & software for further information.

Data

Policy information about availability of data

All manuscripts must include a data availability statement. This statement should provide the following information, where applicable:

- Accession codes, unique identifiers, or web links for publicly available datasets
- A description of any restrictions on data availability
- For clinical datasets or third party data, please ensure that the statement adheres to our policy

Data availability
Numerical source data underlying all graphs can be found in Supplementary data files 1 and 2. Uncropped blots can be found in Supplementary data 3. Additional datasets analysis generated during this study are available on reasonable request.

Research involving human participants, their data, or biological material

Policy information about studies with human participants or human data. See also policy information about sex, gender (identity/presentation), and sexual orientation and race, ethnicity and racism.

Reporting on sex and gender	N/A
Reporting on race, ethnicity, or other socially relevant groupings	N/A
Population characteristics	N/A
Recruitment	N/A
Ethics oversight	N/A

Note that full information on the approval of the study protocol must also be provided in the manuscript.

Field-specific reporting

Please select the one below that is the best fit for your research. If you are not sure, read the appropriate sections before making your selection.

☒ Life sciences ☐ Behavioural & social sciences ☐ Ecological, evolutionary & environmental sciences

For a reference copy of the document with all sections, see nature.com/documents/nr-reporting-summary-flat.pdf

Life sciences study design

All studies must disclose on these points even when the disclosure is negative.

Sample size	No calculation for sample size determination was performed. Sample size was determined to reflect
Data exclusions	No data were excluded from the analysis.
Replication	All data represent at least 3 independent replicates, as indicated in the Figure or Figure legends or Material & Methods.
Randomization	For the in vivo TAA-induced liver fibrosis study in mice, animals were randomized into 5 groups. Details are indicated in the Material & Methods.
Blinding	*Describe whether the investigators were blinded to group allocation during data collection and/or analysis. If blinding was not possible, describe why OR explain why blinding was not relevant to your study.*

Reporting for specific materials, systems and methods

We require information from authors about some types of materials, experimental systems and methods used in many studies. Here, indicate whether each material, system or method listed is relevant to your study. If you are not sure if a list item applies to your research, read the appropriate section before selecting a response.

Materials & experimental systems

n/a	Involved in the study	
☐	☒	Antibodies
☐	☒	Eukaryotic cell lines
☒	☐	Palaeontology and archaeology
☐	☒	Animals and other organisms
☒	☐	Clinical data
☒	☐	Dual use research of concern
☒	☐	Plants

Methods

n/a	Involved in the study	
☒	☐	ChIP-seq
☐	☒	Flow cytometry
☒	☐	MRI-based neuroimaging

Antibodies

Antibodies used	For Western blots, primary and HRP-conjugated secondary antibodies were obtained 1) from Cell Signaling Technology: anti-beta-Actin (4970), anti-Akt (2920), anti-Phospho-Akt S473 (4060), anti-AMPK (2793), anti-Phospho-AMPK T172 (50081), anti-Cofilin S3 (5175), anti-Phospho-Cofilin (3313), anti-p70 S6K (34475), anti-Phospho-S6K T389 (9205), anti-mTOR (2972), anti-Phospho-mTOR

	S2448 (2971), anti-α-Smooth Muscle Actin (SMA; 56856), anti-STAT3 (12640), anti-Phospho-STAT3 Y705 (9145), anti-Phospho-STAT5 Y694 (4322), HRP-conjugated anti-Rabbit IgG (7074), and HRP-conjugated anti-Mouse IgG (7076) ; 2) from Sigma Aldrich: anti-ROCK1 (HPA007567) and anti-ROCK2 (HPA007459); or 3) from eBioscience: CD185 (CXCR5) (12-1859-42), CD279 (PD-1) (11-9969-42), Foxp3 (12-4777-42), CD4 (11-0049-42) and Fixable Viability Dye (65-0866-14).
Validation	All antibodies have been validated according to the manufacturers' information.

Eukaryotic cell lines

Policy information about cell lines and Sex and Gender in Research

Cell line source(s)	Human lung fibroblast MRC-5 cell (CCL-171) was purchased from American Type Culture Collection (ATCC)<br>Murine 3T3L1 (SP-L1-F) cells were purchased from ZenBio Inc.
Authentication	None of cell lines used in this study were authentication
Mycoplasma contamination	Cell lines were not tested for mycoplasma
Commonly misidentified lines<br>(See ICLAC register)	none

Animals and other research organisms

Policy information about studies involving animals; ARRIVE guidelines recommended for reporting animal research, and Sex and Gender in Research

Laboratory animals	Mice, C57BL/6
Wild animals	No wild animal has been used in the study.
Reporting on sex	Female mice were used. It is reported in the Material and Methods.
Field-collected samples	N/A
Ethics oversight	This study was performed at Aragen Bioscience under the Animal Use Protocol: AUP#: 18-0803-MR

Note that full information on the approval of the study protocol must also be provided in the manuscript.

Flow Cytometry

Plots

Confirm that:

☒ The axis labels state the marker and fluorochrome used (e.g. CD4-FITC).

☒ The axis scales are clearly visible. Include numbers along axes only for bottom left plot of group (a 'group' is an analysis of identical markers).

☒ All plots are contour plots with outliers or pseudocolor plots.

☒ A numerical value for number of cells or percentage (with statistics) is provided.

Methodology

Sample preparation	Peripheral blood human CD4+ T cells were purified from the peripheral blood (Leukopak) of healthy human donors between ages of 16 and 75 years (New York Blood Center, NY)
Instrument	Millipore Guava easyCyte 8HT flow cytometer
Software	FlowJo
Cell population abundance	No sorting was performed in this study
Gating strategy	The gating was performed on viable cells by using the viability dye

☒ Tick this box to confirm that a figure exemplifying the gating strategy is provided in the Supplementary Information.
